# EFFECTS OF CIRCADIAN MISALIGNMENT ON BEHAVIOR IN MAPT HUMANIZED MOUSE MODEL

**DOI:** 10.1093/geroni/igae098.2251

**Published:** 2024-12-31

**Authors:** Mesomachi Udenta, Andrea Gonzalez, Qing Lin, Kristi Dieterk, Kevin Koronowski, Juan Palavicini

**Affiliations:** Howard University, Owings mills, Maryland, United States; UT Health, San Antonio, Texas, United States; UT Health, San Antonio, Texas, United States; UT Health, San Antonio, Texas, United States; UT Health, San Antonio, Texas, United States; UT Health SA, San Antonio, Texas, United States

## Abstract

The circadian rhythm is a natural biological process that regulates a multitude of processes including sleep-wake cycles, eating cycles and activity cycles. Circadian misalignment has been found to be related to many neurodegenerative diseases, one of them being Alzheimer’s Disease. Tauopathies, are a distinctive feature of Alzheimer’s Disease and other dementia related disorders. Tau is a protein that helps stabilize he internal structure of brain cells by supporting the microtubules needed for cell structure that oversee maintaining the integrity of the cell. Neurofibrillary tangles are formed when tau accumulates inside the neurons through a process called hyperphosphorylation. These tangles block the neurons transport system and effects the synaptic communication between the neurons which leads to cell damage and eventually cell death. This is the pathway that causes a lot of the major symptoms in Alzheimer’s Disease. In this project. we a MAPT mouse model was used conjunction with a chronic jet lag simulation. The MAPT Gene Replacement Mouse Model consists of genetically modifying mice to express human tau. The model replaces the mouse tau gene with the human tau gene. It can mimic the tau pathology that is seen in humans and allows mice to develop the tau related abnormalities that is seen in dementia related disorders. Studying how this is affected by circadian misalignment can help reveal how sleep wake cycles can influence tau pathology.

